# Prevalence and correlates of suicidal ideation in the general public during the fifth wave of COVID-19 pandemic in Hong Kong

**DOI:** 10.3389/fpsyt.2023.1252600

**Published:** 2024-01-10

**Authors:** Heidi Ka Ying Lo, Joe Kwun Nam Chan, Corine Sau Man Wong, Gabbie Hou Sem Wong, Janet Hiu Ching Lei, Yuen Kiu So, Vivian Shi Cheng Fung, Ryan Sai Ting Chu, Rachel Ling, Albert Kar Kin Chung, Krystal Chi Kei Lee, Calvin Pak Wing Cheng, Wai Chi Chan, Wing Chung Chang

**Affiliations:** ^1^Department of Psychiatry, School of Clinical Medicine, LKS Faculty of Medicine, The University of Hong Kong, Pokfulam, Hong Kong SAR, China; ^2^School of Public Health, LKS Faculty of Medicine, The University of Hong Kong, Pokfulam, Hong Kong SAR, China; ^3^State Key Laboratory of Brain and Cognitive Sciences, The University of Hong Kong, Pokfulam, Hong Kong SAR, China

**Keywords:** suicidal ideation, COVID-19, survey, coping strategies, pre-existing mental disorders, loneliness

## Abstract

**Introduction:**

Literature reveals increased suicidal ideation in the general population during pandemic. However, few COVID-19 studies comprehensively assessed factors associated with suicidal ideation, and mixed findings were observed. We aimed to examine prevalence and correlates of suicidal ideation in general public during the peak of fifth COVID-19 wave in Hong Kong based on a broad array of relevant measures.

**Methods:**

This survey assessed 14,709 community-dwelling adults during March 15–April 3, 2022. Comprehensive assessment was administered including socio-demographics, pre-existing mental/physical morbidity, mental-health symptoms, resilience, loneliness, coping strategies, and pandemic-related factors. Presence of suicidal ideation was evaluated by ratings of item 9 on Patient-Health-Questionnaire-9.

**Results:**

A total of 2,249 (15.3%) participants exhibited suicidal ideation. Multivariable-regression analysis found that being single and unemployed, pre-existing mental disorder, more severe depressive and anxiety symptoms, higher levels of loneliness and engagement in avoidant coping were significantly associated with suicidal ideation. Conversely, attaining tertiary educational level or above, greater resilience and adopting problem-focused coping were associated with lower likelihood of suicidal ideation. Although univariate-analyses revealed that a number of pandemic-related factors were linked to suicidal ideation, none remained significant in the multivariable model.

**Conclusion:**

A significant proportion of people experienced suicidal ideation during the peak of fifth COVID-19 wave. Risk and protective factors identified would facilitate early identification of high-risk individuals and provision of targeted interventions to minimize suicidal ideation and risk of self-harm. Caution should be exercised due to study limitations of a cross-sectional design which precluded establishing causality among variables, and reliance on self-reported data.

## Introduction

Coronavirus disease (COVID-19) was declared a global pandemic by the World Health Organization in March 2020 (1), for imposing public health crisis. COVID-19 literature conducted in the community consistently demonstrated its profound impacts on both physical and mental health outcomes, including anxiety, depressive symptoms and psychological distress (2, 3). The effects of the pandemic itself combined with the prolonged implementation of social containment policies led to indirect socioeconomic repercussions, including economic downturn (4), social isolation (5), unemployment (6), and barriers to access mental health services (7) due to public health policies might in turn increase suicidality (8).

An elevated risk of suicide has been consistently linked to pandemic outbreaks across the world (9), with increased completed suicide rate in the United States during the 1918–1919 influenza pandemic (10), and increased rate of suicidal attempts during Ebola outbreak (11). Similarly, increased likelihood of suicide behaviors were observed during the COVID-19 pandemic (12–14). Hong Kong (HK) was the epicentre of the Severe Acute Respiratory Syndrome (SARS) in 2003, which caused 1,755 infections and 300 fatalities (15), and endured months of social unrest in 2019 just before the first local outbreak of COVID-19. Previous studies found an increased suicide rate in the elderlies during SARS epidemic in HK (15, 16). Throughout the whole 2021, there were fewer than 4,000 COVID-19 cases reported in HK (17). HK then experienced the fifth COVID-19 wave after the emergence of the Omicron variant in December 2021, with the 7-day rolling average of COVID-related deaths reaching 3.73 per 1,000 people at the peak, the highest worldwide (18, 19). However, until now, there has been lack of research evaluating suicidal ideations in the general population of HK that had this distinctive pandemic experience.

Suicidal ideation is defined as thinking about, considering or planning suicide (20). It is critical to understand suicidal ideation, which is a strong predictor of subsequent suicide attempts and completed suicides (21). Detecting its prevalence, risk factors and protective factors in the general population is essential for informing effective suicide prevention strategies. Development of suicidal ideation is multifactorial and involves a complex interplay between biological and psychosocial determinants (22). Some sociodemographic characteristics (such as younger age, socioeconomic disadvantage, single marital status and unemployment) and COVID-19 pandemic-related factors (such as contracting COVID-19 and social containment measures) are reported to be associated with suicidal ideations (23–27). Patients with pre-existing mental disorders are more likely than healthy persons to suffer an emotional response to the COVID-19 pandemic, leading to relapse or worsening of symptoms (7). For instance, people with a pre-existing mood or anxiety disorder had considerably higher scores of COVID-19-related traumatic stress symptoms than individuals without a current mental condition (28). Furthermore, while previous studies have consistently reported loneliness as a significant risk factor for suicidal ideation (24, 29–31), there is a gap in the literature simultaneously encompassing other protective psychosocial measures [e.g., different coping styles and resilience that have been linked to improved mental health outcomes in other contexts (32)] in studying suicidal ideations during COVID-19 pandemic. Relatively few studies have been conducted to systematically evaluate a broad range of factors associated with suicidal ideations in the general population during the COVID-19 pandemic.

To this end, the current study aimed to assess the prevalence of suicidal ideation among the general population amidst the peak of the fifth COVID-19 wave in HK (March–April, 2022). Importantly, we examined the correlates of suicidal ideation across a comprehensive array of variables encompassing socio-demographics, pre-existing illness profile, mental health symptoms, psychosocial measures as well as pandemic-related factors.

## Materials and methods

### Participants and study setting

This cross-sectional survey aimed to recruit community-dwelling adults aged 18 years or above in HK from March 15 to April 3, 2022 (33), during which the city was experiencing the peak of the fifth COVID-19 wave. It was estimated that approximately half of the population (3.6 million) have contracted the infection during the fifth wave by mid-March (34), and public health containment and social-distancing measures were further tightened in response to the surge of COVID cases (17). Details of the study design have been described in our recently published report (33), which examined the association between COVID-19 perseverative cognition and depression, and the moderating effect of potential risk and protective factors on this association in the general public. Briefly, we adopted snowballing sampling technique for data collection, with an online anonymous self-rated questionnaire being administered on a Qualtrics survey platform.^1^ Virtual snowballing sampling is a commonly-used recruitment strategy during COVID-19 pandemic [e.g., (35–37)], which enables researchers to obtain a large sample within a short period of time and to minimize the interference of stringent control measures on data collection. The survey was disseminated through social media platforms (e.g., Facebook, Instagram, Twitter, WhatsApp), universities (via emails), and the Hong Kong Public Opinion Research Institute (HKPORI) which sent email invitations with survey link to the members of its probability- and non-probability- based online panels of adult HK residents. HKPORI is a well-established survey agency which has executed numerous independent public surveys for academic institutions and government departments in HK (38). Respondents were encouraged to forward the survey link to their social networks for study participation. Survey participation was on a voluntary basis and online informed consent was obtained before questionnaire assessment. The survey took around 20 min to complete. The study was approved by the Institutional Review Board of the University of Hong Kong/Hospital Authority Hong Kong West Cluster (HKU/HA HKW). The survey received 20,971 responses. Respondents who were not residing in HK during the study period, did not provide consent or failed to complete the questionnaire items on the primary measure of the current study (i.e., the Patient Health Questionnaire [PHQ-9] including the last item which assessed self-reported suicidal ideation) were excluded. A total of 14,709 respondents constituted the final sample for the current analysis.

### Assessments

The self-rated study assessment comprised four sections including sociodemographic, illness profile and mental health symptoms, psychosocial measures, and COVID-19-related factors. Sociodemographic characteristics included age, gender, marital status, highest attained educational level, employment status, size of residential housing and monthly household income. Illness profile included pre-existing mental health diagnosis, alcohol/substance use, and chronic physical disease.

Concerning mental health symptoms, depressive and anxiety symptom severity were assessed by Patient Health Questionnaire-9 [PHQ-9; (39, 40)] and Generalized Anxiety Disorder-7 scale [GAD-7; (41, 42)], respectively, with both scales using a 4-point Likert scale ranging from 0 (never) to 3 (nearly every day). A validated Chinese version of Obsession with COVID-19 Scale [OCS; (43, 44)] was administered to measure the frequency of persistent and disturbing thinking about COVID-19, within a 2-week timeframe on a 5-point Likert scale ranging from 0 (not at all) to 4 (nearly every day). In the current study, depressive symptom severity was quantified by the sum of the items 1 to 8 on PHQ-9. Sleep quality and disturbance were assessed using Insomnia Severity Index [ISI; (45, 46)]. For all of the symptom scales, higher scores indicated greater symptom severity. Following the method of previous studies examining suicidal ideations in general public during COVID-19 (26, 47), an item 9 on the PHQ-9, namely “During the past 2 weeks, how often have you been troubled by the notion that you would be better off dead or hurting yourself?,” was used to evaluate the presence of suicidal ideation. Specifically, participants who responded to an item 9 as “not at all” (i.e., rating = 0) were considered having no suicidal ideation, whereas those whose rating was 1–3 (i.e., “several days,” “more than half the days,” or “nearly every day”) were categorized as having suicidal ideation.

Regarding psychosocial measures, participants’ coping strategies were assessed by an adapted Coping Orientation to Problems Experienced Inventory–Brief [Brief-COPE; (48, 49)], which used a 4-point Likert scale ranging from 0 (never) to 3 (always). The 14 items of the adapted Brief-COPE were grouped into 3 copying styles based on previous factor-analytic study (50), namely avoidant, emotion-focused and problem-focused coping styles for subsequent analysis. A higher item sum score indicated a higher level of engagement in that particular coping style. The Chinese version of the three-item UCLA Loneliness Scale was applied to measure loneliness on a three-factor structure (51, 52). The Brief Resilience Scale [BRS; (53, 54)] was used to assess resilience levels on a 5-point Likert scale (1 [strongly disagree] to 5 [strongly agree]), with higher scores indicating greater resilience. Evaluation of COVID-19-related factors comprised items assessing history of contracting COVID-19 infection, receipt of vaccination, exposure to COVID-19-related information, COVID-19-related stressors experienced, specific infection control measures (e.g., under quarantine, mandatory COVID-19 testing) experienced and associated distress. Details of assessment of COVID-19 related factors are summarized in Supplementary Table S1.

### Statistical analysis

First, a series of univariate binary logistic regression analyses were conducted to examine the association of suicidal ideation status (yes vs. no) with a broad array of variables covering socio-demographics, illness profiles and mental health symptoms, psychosocial measures and COVID-19 related factors. Then, those variables that were found to be statistically significant in the preceding univariate analyses were then included in the multivariable binary logistic regression model, based on backward Wald statistics, to determine factors which were independently associated with suicidal ideation. All analyses were conducted using SPSS 23.0, with significance level set as *p* < 0.05.

## Results

### Sample characteristics and prevalence of suicidal ideation

Table 1 summarizes the characteristics of the study sample. A total of 52.9% participants were women, and 14.3, 16.0, 17.4, 19.2 and 33.1% were aged 18–29, 30–39, 40–49, 50–59, and ≥60 years, respectively. Slightly more than half of the sample (56.4%) were married, and 35% attained tertiary educational level. Based on the ratings of item 9 on PHQ-9, 15.3% (*n* = 2,249) participants were classified as having suicidal ideation, including 10.7% (*n* = 1,570) seldom reported suicidal ideation, 2.8% (*n* = 413) often had suicidal ideation and 1.8% (*n* = 266) always reported suicidal ideation over the past 2 weeks of survey assessment.

**Table 1:** Descriptive characteristics of the study sample.

Variables of interest	Number (%)/mean (SD)
Socio-demographics	
Gender, female	7,653 (52)
Age, years	
18–29	2,100 (14.3)
30–39	2,346 (16.0)
40–49	2,563 (17.4)
50–59	2,823 (19.2)
60 or above	4,876 (33.1)
Education	
Secondary level or below	9,554 (65.0)
Tertiary level or above	5,153 (35.0)
Marital status	
Single	5,023 (34.2)
Married	8,296 (56.4)
Divorced/widowed	1,388 (9.4)
Employment status	
Working/student/housewife	10,344 (70.3)
Unemployed	1,090 (7.4)
Retired	3,272 (22.3)
Size of residential housing, square feet	
Less than 300	2,727 (18.8)
301–500	5,066 (34.9)
Above 501	6,743 (46.3)
Household income (HKD, monthly)^a^	
25,000 or below	6,711 (46.2)
Above 25,000	7,805 (53.8)
Illness profile and mental health symptoms	
Chronic physical diseases, yes	4,887 (33.7)
Alcohol/ substance abuse, yes	683 (4.7)
Pre-existing mental disorder, yes	1,205 (8.3)
Depressive symptoms (PHQ-9)	6.37 (5.6)
Anxiety symptoms (GAD-7)	5.58 (5.5)
Perseverative cognition on COVID-19 (OCS)	4.17 (3.6)
Insomnia symptoms (ISI)	8.38 (6.0)
Psychosocial measures	
Avoidant coping style	10.43 (2.8)
Emotion-focused coping style	10.22 (3.0)
Problem-focused coping style	6.37 (2.1)
Loneliness	4.46 (1.7)
Resilience	19.73 (4.6)
COVID-19 related factors	
Distress from experiencing the tightening of social-distancing measures	5.21 (3.5)
History of COVID-19 infection, yes	3,496 (23.8)
COVID-19 vaccination received	
0–2 doses	8,058 (56.8)
3 doses	6,124 (43.2)
Mandatory testing/ quarantine, yes	4,360 (31.4)
Number of COVID-19 related stressors	
0–2	11,235 (79.4)
3–5	2072 (14.6)
6–8	841 (6.0)
Time spent on reading COVID-19 related information	
<1 h per day	7,095 (49.9)
1–3 h per day	5,288 (37.2)
≥4 h per day	1843 (12.9)

COVID, coronavirus; GAD-7, Generalized Anxiety Disorder-7 scale; HKD, Hong Kong dollars; ISI, Insomnia Severity Index; OCS, Obsession with COVID-19 Scale; PHQ-9, Patient Health Questionnaire-9; SD, standard deviation.^a^ As of 9th March 2023, 1HKD = 0.13 USD.

### Correlates of suicidal ideation

As shown in Table 2, participants’ age, educational level, marital status, employment status, size of residential housing and monthly household income were significantly related to suicidal ideation in univariate regression analyses. Regarding illness profile and mental health symptoms, ratings on PHQ-9, GAD-7, OCS and ISI were associated with suicidal ideation. Table 3 shows that UCLA Loneliness Scale scores, BRS scores, and higher levels of engagement with avoidant and problem-focused coping styles were significantly related to suicidal ideation. For pandemic-related factors, history of COVID-19 infection, vaccination status, history of mandatory testing/quarantine, number of COVID-19 related stressors, exposure on reading pandemic-related information, and level of distress due to social-distancing measures were associated with suicidal ideation in univariate regression analyses.

**Table 2:** Univariate regression analyses on the relationship of suicidal ideation with socio-demographics, illness profile and mental health symptoms.

Variables of interest^a^	No suicidal ideation	Suicidal ideation	Odds ratio (95% CI)	Value of *p*
Socio-demographics				
Age, years				
18–29	1,557 (12.5)	544 (24.2)	1.00 (reference)	–
30–39	1865 (15.0)	482 (21.4)	0.74 (0.64–0.85)	<0.001
40–49	2,144 (17.2)	419 (18.6)	0.56 (0.49–0.65)	<0.001
50–59	2,418 (19.4)	405 (18.0)	0.48 (0.42–0.55)	<0.001
≥60	4,476 (35.9)	400 (17.8)	0.26 (0.22–0.30)	<0.001
Gender (male)	5,818 (47.4)	993 (45.5)	1.00 (reference)	–
Gender (female)	6,464 (52.6)	1,189 (54.5)	1.08 (0.98–1.18)	0.112
Education				
Secondary level or below	7,901 (63.4)	1,653 (73.5)	1.00 (reference)	–
Tertiary level or above	4,557 (36.6)	595 (26.5)	0.62 (0.57–0.69)	<0.001
Marital status				
Married/stable relationship	7,362 (59.1)	934 (41.5)	1.00 (reference)	–
Single	3,965 (31.8)	1,059 (47.1)	2.11 (1.91–2.31)	<0.001
Divorced/ widowed	1,132 (9.1)	256 (11.4)	1.78 (1.53–2.07)	<0.001
Employment status				
Working/ students/housewife	8,676 (69.6)	1,668 (74.1)	1.00 (reference)	<0.001
Unemployed	790 (6.4)	301 (13.4)	1.98 (1.71–2.28)	<0.001
Retired	2,992 (24.0)	280 (12.5)	0.49 (0.43–0.56)	<0.001
Size of residential housing, square feet				
Less than 300	2,104 (17.1)	624 (28.0)	1.00 (reference)	–
301–500	4,289 (34.8)	777 (34.9)	0.61 (0.54–0.69)	<0.001
Above 501	5,918 (48.1)	825 (37.1)	0.47 (0.42–0.53)	<0.001
Household income (HKD, monthly)^b^				
25,000 or below	5,516 (44.8)	1,195 (53.7)	1.00 (reference)	–
Above 25,000	6,775 (55.2)	1,030 (46.3)	0.70 (0.64–0.77)	<0.001
Illness profile and mental health symptoms				
Chronic physical diseases, no	8,294 (67.4)	1,336 (60.2)	1.00 (reference)	
Chronic physical diseases, yes	4,003 (32.6)	884 (39.8)	1.37 (1.25–1.50)	<0.001
Alcohol/ substance abuse, no	11,827 (96.2)	2007 (90.4)	1.00 (reference)	
Alcohol/ substance abuse, yes	471 (3.8)	213 (9.6)	2.66 (2.25–3.15)	<0.001
Pre-existing mental disorder, no	11,556 (94.0)	1756 (79.1)	1.00 (reference)	
Pre-existing mental disorder, yes	741 (6.0)	464 (20.9)	4.12 (3.63–4.67)	<0.001
Depressive symptoms (PHQ-9)^c^	5.2 (4.6)	13.3 (5.7)	1.29 (1.28–1.30)	<0.001
Anxiety symptoms (GAD-7)	4.5 (4.6)	12.1 (5.7)	1.27 (1.25–1.28)	<0.001
Perseverative cognition on COVID-19 (OCS)	3.8 (3.4)	6.0 (4.1)	1.17 (1.16–1.19)	<0.001
Insomnia symptoms (ISI)	7.4 (5.4)	13.7 (6.3)	1.18 (1.17–1.19)	<0.001

CI, confidence interval; COVID, coronavirus; GAD, generalized anxiety disorder scale; HKD, Hong Kong dollars ISI Insomnia Severity Index; OCS, obsession with COVID-19 scale; PHQ, patient health questionnaire; SD, standard deviation.^a^ Variables of interest are presented in number and percentage (%) except scores on PHQ-9, GAD-7, OCS and ISI, which are presented in mean and standard deviation.^b^ As of 9th March 2023, 1HKD = 0.13 USD.^c^ Depressive symptom severity was quantified as the sum of ratings from item 1 to 8 on PHQ-9, excluding item 9, which measured suicidal ideation.

**Table 3:** Univariate regression analyses on the relationship of suicidal ideation with psychosocial measures and COVID-19 related factors.

Variables of interest^a^	No suicidal ideation (*n* = 12,460)	Suicidal ideation (*n* = 2,249)	Odds ratio (95% CI)	Value of *p*
Psychosocial measures				
Avoidant coping style	10.0 (2.5)	12.7 (3.1)	1.42 (1.39–1.45)	<0.001
Emotion-focused coping style	10.2 (3.0)	10.2 (2.7)	1.00 (0.98–1.01)	0.492
Problem-focused coping style	6.8 (2.1)	6.6 (1.9)	1.06 (1.04–1.08)	<0.001
Loneliness	4.2 (1.5)	6.0 (2.0)	1.72 (1.68–1.77)	<0.001
Resilience	20.5 (4.2)	15.7 (4.2)	0.77 (0.76–0.78)	<0.001
COVID-19 related factors				
Distress due to social-distancing measures	4.98 (3.4)	6.49 (3.3)	1.14 (1.13–1.16)	<0.001
Never have COVID-19 infection	9,557 (76.7)	1,656 (74.0)	1.00 (reference)	–
History of COVID-19 infection, yes	2,903 (23.3)	582 (26.0)	1.18 (1.06–1.30)	0.002
COVID-19 vaccination doses received				
0–2 doses	6,688 (55.5)	1,382 (64.0)		
3 doses	5,357 (44.5)	778 (36.0)	0.70 (0.64–0.77)	<0.001
Mandatory testing/ quarantine, no	8,151 (69.3)	1,356 (64.5)	1.00 (reference)	–
Mandatory testing/ quarantine, yes	3,615 (30.7)	745 (35.5)	1.24 (1.12–1.37)	<0.001
Number of COVID-19 related stressors				
0–2	10,191 (84.9)	831 (38.5)	1.00 (reference)	–
3–5	1,408 (11.7)	857 (39.8)	4.52 (4.04–5.05)	<0.001
6–8	408 (3.4)	468 (21.7)	10.2 (8.8–11.8)	<0.001
Time spent on reading COVID-19 related information				
<1 h per day	6,264 (51.9)	842 (38.7)	1.00 (reference)	–
1–3 h per day	4,431 (36.7)	861 (39.5)	1.45 (1.30–1.60)	<0.001
≥4 h per day	1,375 (11.4)	475 (21.8)	2.57 (2.27–2.92)	<0.001

CI, confidence interval; COVID, coronavirus.^a^ All of the variables of psychosocial measures and distress due to social-distancing measures are presented in mean and standard deviation, while the remaining variables of COVID-19 related factors are presented in number and percentage (%).

A multivariable logistic regression analysis revealed that attainment of tertiary educational level or above, greater resilience, and adoption of problem-focused coping style were independently associated with decreased likelihood of experiencing suicidal ideation (Table 4), while the relationship between older age (≥60 years) and suicidal ideation approached statistical significance (*p* = 0.056). Conversely, single marital status, being unemployed, pre-existing mental disorder, more severe depressive and anxiety symptoms, higher levels of loneliness, and engagement in avoidant coping style were associated with increased risk of suicidal ideation.

**Table 4:** Multivariable logistic regression analysis for factors associated with suicidal ideation.

Variables	Odds ratio (95% CI)	*Value of p*
Socio-demographics		
Age, years		
18–29	1.00 (reference)	–
30–39	0.98 (0.81–1.20)	0.86
40–49	0.90 (0.73–1.11)	0.32
50–59	1.06 (0.85–1.33)	0.61
≥ 60	0.79 (0.61–1.01)	0.056
Education		
Secondary level or below	1.00 (reference)	–
Tertiary level or above	0.80 (0.70–0.91)	<0.001
Marital status		
Married/ stable relationship	1.00 (reference)	–
Single	1.33 (1.14–1.54)	<0.001
Divorced/widowed	1.20 (0.98–1.47)	0.085
Employment Status		
Working/students/housewife	1.00 (reference)	–
Unemployed	1.31 (1.08–1.60)	0.006
Retired	1.20 (0.95–1.52)	0.126
Size of residential housing, square feet		
Less than 300	1.00 (reference)	
301–500	0.89 (0.75–1.04)	0.148
Above 501	0.92 (0.78–1.10)	0.356
Household income (HKD, monthly)		
25,000 or below	1.00 (reference)	
Above 25,000	0.96 (0.84–1.10)	0.589
Illness profile and mental health symptoms		
Chronic physical diseases, no	1.00 (reference)	–
Chronic physical diseases, yes	0.90 (0.77–1.05)	0.187
Alcohol/ substance abuse, no	1.00 (reference)	–
Alcohol/ substance abuse, yes	1.09 (0.86–1.40)	0.470
Pre-existing mental disorder, no	1.00 (reference)	–
Pre-existing mental disorder, yes	1.51 (1.23–1.86)	<0.001
Depressive symptoms	1.14 (1.12–1.16)	<0.001
Anxiety symptoms	1.08 (1.06–1.10)	<0.001
Perseverative cognition on COVID-19	0.99 (0.97–1.01)	0.340
Insomnia symptoms	1.00 (0.99–1.02)	0.344
Psychosocial measures		
Avoidant coping style	1.13 (1.10–1.16)	<0.001
Problem-focused coping style	0.88 (0.85–0.91)	<0.001
Loneliness	1.15 (1.11–1.19)	<0.001
Resilience	0.92 (0.91–0.94)	<0.001
COVID-19 related factors		
Distress due to social-distancing measures	1.01 (0.99–1.03)	0.328
Never have COVID-19 infection	1.00 (reference)	–
History of COVID-19 infection, yes	1.04 (0.90–1.19)	0.608
COVID-19 vaccination doses received		
0–2 doses	1.00 (reference)	–
3 doses	0.90 (0.79–1.02)	0.091
Mandatory testing/ quarantine, no	1.00 (reference)	–
Mandatory testing/ quarantine, yes	1.08 (0.96–1.23)	0.196
Number of COVID-19 related stressors		
0–2	1.00 (reference)	–
3–5	1.08 (0.92–1.25)	0.357
6–8	1.18 (0.95–1.47)	0.144
Time spent on reading COVID-19 related information		
<1 h per day	1.00 (reference)	–
1–3 h per day	1.00 (0.88–1.15)	0.957
≥4 h per day	1.01 (0.84–1.22)	0.920

CI, confidence interval; COVID, coronavirus; HKD, Hong Kong dollars.

## Discussion

The current study aimed to examine the prevalence and correlates of suicidal ideation in the general population (predominantly Chinese) during the peak of the fifth COVID-19 wave in HK. This is among the few studies that comprehensively assessed a wide array of factors associated with suicidal ideation in the general public amidst the pandemic, encompassing socio-demographics, illness profile and mental health symptoms, psychosocial variables of loneliness, resilience and coping strategies, as well as COVID-19 related variables. Our results showed that approximately one-sixths (15.3%) of the study sample experienced suicidal ideation amidst the fifth wave of pandemic. This is slightly higher than the pooled prevalence estimate of suicidal ideation during COVID-19 reported in a recent meta-analysis (26), as well as those observed in the Chinese population from mainland China (13.4%) and Taiwan (10.8%) (26, 54). The distinctive experiences of HK population with unique prior exposure of the SARS epidemic and social unrest just before COVID-19 may have also contributed to the susceptibility to increased suicidal ideation during the fifth wave of pandemic.

Our findings are generally consistent with the notion of the complex interplay among various risk and protective factors for suicidal ideation (22). We affirmed results of previous COVID-19 studies that a number of key socio-demographic characteristics were significantly associated with increased risk of suicidal ideation (6, 23–26). Being single, unemployed and less educated are well-established risk factors for suicidal ideation and behaviors in the literature, irrespective of the pandemic. Alternatively, our observation that aged ≥60 years was related to the lowest likelihood of having suicidal ideation (albeit of trend-wise significance) suggested that people of older age might be relatively susceptible to the development of suicidal ideation during pandemic. This in fact corroborated with some studies examining suicidal ideation during the COVID-19 using online survey design (23, 25, 26). Of note, we are cautious that web-based survey data may potentially bias the results in favor of those more resourceful older-aged participants (among the elderly population) who had access to online platforms and were equipped with the skills to administer the survey questionnaire (55). This is intriguing given the contrary finding that elderly people, but not the younger-age group, had an increased suicide rate during SARS epidemic in HK (15, 16). Alternatively, it is possible that compared to SARS, COVID-19 pandemic is far more long-lasting with more prolonged public health and social-distancing measures for infection control. Yet, older-aged people are in general less likely to be employed or engaged in activities that require frequent face-to-face interactions. Hence, they may be comparatively less affected by the stringent public health polices, with lower degree of persistent exposure to COVID-19 related stressors such as unemployment and financial uncertainty, and the consequent adverse psychological impact including suicidal ideation.

We found that more severe depressive and anxiety symptoms, and the presence of pre-existing mental disorder were independently associated with raised likelihood of developing suicidal ideation. Substantial evidence has noted that individuals with mental disorders are more vulnerable to increased psychological distress than those without mental disorders during the pandemic (7, 28). It is also well-acknowledged that mental disorders represent a key predictor of suicidal ideation and behaviors (56, 57). Consistent with prior research (25), our findings demonstrated that pre-existing mental disorder was the strongest factor associated with suicidal ideation in the general population during the fifth COVID-19 wave. In line with a large body of research (and disregard of the pandemic) (27, 47, 55, 58, 59), we observed that greater depressive and anxiety symptom severity were significantly related to suicidal ideation. Accumulating data have further suggested that these mood symptoms may even persist after the pandemic (60). Overall, our results highlight the significance of early identification of and prompt treatment to depressive and anxiety symptoms, as well as the need to optimize psychiatric treatment for pre-existing mental disorders so as to minimize the risk of suicidal ideation and subsequent self-harm attempts during the pandemic. In this regard, health and welfare polices ensuring adequate access and availability of mental health services and mobilizing community service support would facilitate early detection of high-risk subgroup and timely provision of effective intervention to alleviate mood symptoms and reduce self-harm risk (61). Emerging evidence has also demonstrated the effectiveness of tele-health care including mental health screening, assessment and treatment of depression (62), and indicated this approach as a feasible and reliable alternative of standard healthcare delivery during pandemic (63, 64). Future research should concentrate on evaluating tailor-made mental health interventions to meet the specific need of this high-risk subgroup, who have pre-existing mental disorders and/or persistent mood symptoms, during and after the pandemic (7).

Our results demonstrated that greater resilience and higher level of engagement in problem-focused coping style were significantly associated with lower risk of developing suicidal ideation during the peak of fifth pandemic wave. Alternatively, higher degree of loneliness and adoption of avoidant coping style were linked to suicidal ideation. This generally concurs with the research conducted before COVID-19 showing that avoidant coping strategies are a risk factor for suicidal ideation (65), whereas problem-focused coping can be protective from self-harm (66, 67). Resilience is the capacity and dynamic process of adaptively overcoming stress and adversity while maintaining normal functioning (68). Higher level of resilience has been found to reduce depressive symptoms (69), mitigate the effect of pandemic-related stress on negative mental health outcomes (70), and to be a protective factor against suicide risk (71, 72). Our finding that loneliness was independently associated with suicidal ideation is in line with previous studies, including those conducted during the COVID-19 pandemic, showing that loneliness is a significant predictor of suicidal ideation and behavior (24, 30, 73). It is noteworthy that loneliness and social connection are separate constructs with overlapping features (74). Loneliness, defined as the subjective perception of insufficient social contact, can exacerbate feelings of vulnerability and heighten hypervigilance to social threats, which adversely affect psychosocial functioning and may result in poorer mental health outcomes (75). In this regard, loneliness may be more closely associated with suicidal ideation and behavior than other aspects of social connection, such as social distancing, emotional support and interpersonal conflict (76). An earlier local COVID-19 study also found that subjective feelings of loneliness were associated with increased mental health symptoms among the general public in HK (77). Taken together, these results underscore the importance of promoting resilience (at individual and community levels, for instance via practicing self-care and cultivating community) (72, 78), social support (i.e., reduction of sense of loneliness) and the use of adaptive coping as effective strategies to minimize the adverse impact of pandemic on increased suicidal ideation and self-harm risk (79). On the other hand, although our univariate analyses revealed that a number of COVID-19 related factors, such as number of pandemic-related stressors, distress due to social-distancing measures, history of contracting COVID-19 and vaccination status, are related to suicidal ideation, none of these variables remained significant in the final multivariable model. This is contrary to some past studies which suggested that certain COVID-19 related factors including experience of quarantine and confirmed infection were significantly related to higher odds of suicidal ideation (23, 26, 27). It might be possible that an effect of COVID-19 related factors on suicidal ideation may be fully mediated by other variables included in our final regression model, which incorporated a comprehensive range of candidate factors encompassing illness profile, mental health symptoms and psychosocial measures. Alternatively, it is important to take into consideration the broader context beyond the impact of the fifth pandemic wave. Previous COVID-19 pandemic waves, the accompanying financial/economic crisis (4), and ensuing geopolitical shifts (80) could also pose influences on individuals’ mental wellbeing and suicidality. These broader-contextual factors can compound the stress and isolation imposed by pandemic circumstances, intensifying feelings of loneliness and vulnerability to psychological distress (81). As such, future investigation should clarify the complex interplay between these factors on mental health outcomes.

The study has several limitations. First, the cross-sectional study design precludes establishment of causal relationship. Longitudinal research is warranted to determine the course of suicidal ideations over time and its predictors. Second, although an item 9 on PHQ-9 is a commonly used measure in psychiatric research to determine the presence of suicidal ideation (21, 26, 47, 82), scales that are specifically developed for suicidal ideation/suicide risk assessment would provide a more comprehensive and accurate evaluation (83). Third, the snowballing sampling strategy is non-probabilistic in nature and may compromise the representativeness of the sample and study results. Additionally, the questionnaire was distributed and administered online, people with limited access to smartphone or computers would be excluded from the survey, resulting in some selection bias. Nevertheless, online health survey with virtual snowballing strategy is a widely-used and reliable method to assess psychological distress in the general population amidst the COVID-19 pandemic, particularly in the face of difficulties to access and recruit participants under the circumstances of stringent community containment measures to curb the spread of infection (84). Fourth, psychological symptom assessment was based on participants’ self-reporting (though these scales are well-validated and commonly used in research), which may not well align with the corresponding rating instruments administered by mental health professionals.

Our study showed that around 15% of the general population in HK displayed suicidal ideation during the peak of the fifth wave of the COVID-19 pandemic. People who are single, less educated and unemployed, have pre-existing mental disorder, higher levels of loneliness, depressive and anxiety symptoms, and engagement of avoidant coping style are significantly more likely to experience suicidal ideation. Conversely, greater resilience and the use of problem-focused coping strategies are protective factors for developing suicidal ideation. Prospective follow-up investigation is required to track the trajectories of suicidal ideation and behaviors as well as their predictors in relation to the subsequent course of COVID-19 pandemic and the post-pandemic era.

## Data availability statement

The raw data supporting the conclusions of this article will be made available by the authors, without undue reservation.

## Ethics statement

The studies involving humans were approved by Institutional Review Board of the University of Hong Kong/Hospital Authority Hong Kong West Cluster (HKU/HA HKW). The studies were conducted in accordance with the local legislation and institutional requirements. The participants provided their written informed consent to participate in this study.

## Author contributions

WiC and CW designed and conceptualized the study. GW, JL, YS, JC, VF, and RC prepared the questionnaire assessment. HL and JC conducted the statistical analysis. HL wrote the first draft of the manuscript. HL, WiC, and JC interpreted the study data. WiC and HL revised and finalized the manuscript. All authors provided critical feedback to the manuscript and have approved the final manuscript.

## Footnotes

^1^ https://www.qualtrics.com
