# Supplementary material for: Prevalence and correlates of suicidal ideation in the general public during the fifth wave of COVID-19 pandemic in Hong Kong
Source: Front Psychiatry. 2024 Jan 10;14:1252600. doi: 10.3389/fpsyt.2023.1252600 (PMC10809397; doi:10.3389/fpsyt.2023.1252600)
Supplement: Supplementary file 1 [file Table_1.docx]

**Table S1** Description of questionnaire items specifically designed for COVID-19 related factors.

| Variables | Item description |
| --- | --- |
| COVID-19 infection | Participants were asked whether or not they had contracted COVID-19 before. |
| COVID-19 vaccine doses received | Participants were asked how many doses of COVID-19 vaccination they have received by choosing “0”, “1”, “2” or “3”. |
| Time spent on reading COVID-19 related information per day | Participants were asked how much they have spent on reading COVID-19 related information on average each day in the past 2 weeks. “None”, “Less than an hour”, “1-3 hours”, “4-6 hours”, “More than 6 hours” were the options. |
| Number of COVID-19 related stressors | Participants were asked the level of COVID-related stress they experienced during the fifth wave in each of the 8 domains, with each domain being assessed using a 5-point Likert scale ranging from 0 (not stressed) to 4 (extremely stressed): (1) financial, (2) work, (3) physical health, (4) mental health, (5) food and supplies, (6) medicine, (7) family relationship, and (8) other interpersonal relationships. A rating of 2 or above in each of the 8 domains would be regarded as a stressor. Level of COVID-related stress was quantified by the total number of stressors. |
| Being imposed on mandatory infection control measures | Participants were asked if they have experienced the following measures during fifth wave of COVID-19. Two measures were selected using a dichotomous (yes-no) scale: (1) Required to undergo mandatory quarantine without being tested positive and (2) Required to undergo mandatory testing as the building you lived in was listed in Restriction-testing Declaration |
| Distress from experiencing the tightening of social-distancing measures ^a^ | Participants were asked to indicate their level of distress from experiencing the tightening of social distancing measures during fifth wave of COVID-19. A 11-point Likert scale (0=Not stressed at all, 10=Extremely stressed) was used. |

^a^ Tightening of social-distancing measures included prohibition of group gathering of more than 2 people in public place, restriction of no more than 2 customers per table in catering premises, dine-in ban after 6 p.m. and closure of all recreational premises.
